# Reducing the impact of intensive care unit mattress compressibility during CPR: a simulation-based study

**DOI:** 10.1186/s41077-017-0057-y

**Published:** 2017-11-16

**Authors:** Yiqun Lin, Brandi Wan, Claudia Belanger, Kent Hecker, Elaine Gilfoyle, Jennifer Davidson, Adam Cheng

**Affiliations:** 1 KidSIM-ASPIRE Simulation Research Program, Alberta Children’s Hospital, University of Calgary, 2888 Shaganappi Trail NW, Calgary, AB T3B 6A8 Canada; 2 Faculty of Nursing, University of British Columbia, T201-2211 Westbrook Mall, Vancouver, BC V6T 2B5 Canada; 3 Faculty of Kinesiology, Queens University, 99 University Ave, Kingston, ON K7L 3N6 Canada; 4 Department of Community Health Sciences, Cumming School of Medicine and Faculty of Veterinary Medicine, University of Calgary, 3330 Hospital Dr NW, Calgary, AB T2N 4N1 Canada; 5 Department of Pediatrics, Section of Critical Care, Cumming School of Medicine, Alberta Children’s Hospital, University of Calgary, 2888 Shaganappi Trail NW, Calgary, AB T3B 6A8 Canada; 6 Division of Emergency Medicine, Department of Pediatrics and KidSIM-ASPIRE Research Program, Alberta Children’s Hospital, 2888 Shaganappi Trail NW, Calgary, AB T3B 6A8 Canada

**Keywords:** Cardiopulmonary resuscitation, Quality, Resuscitation, Chest compressions, Mattress

## Abstract

**Background:**

The depth of chest compression (CC) during cardiac arrest is associated with patient survival and good neurological outcomes. Previous studies showed that mattress compression can alter the amount of CCs given with adequate depth. We aim to quantify the amount of mattress compressibility on two types of ICU mattresses and explore the effect of memory foam mattress use and a backboard on mattress compression depth and effect of feedback source on effective compression depth.

**Methods:**

The study utilizes a cross-sectional self-control study design. Participants working in the pediatric intensive care unit (PICU) performed 1 min of CC on a manikin in each of the following four conditions: (i) typical ICU mattress; (ii) typical ICU mattress with a CPR backboard; (iii) memory foam ICU mattress; and (iv) memory foam ICU mattress with a CPR backboard, using two different sources of real-time feedback: (a) external accelerometer sensor device measuring total compression depth and (b) internal light sensor measuring effective compression depth only. CPR quality was concurrently measured by these two devices. The differences of the two measures (mattress compression depth) were summarized and compared using multilevel linear regression models. Effective compression depths with different sources of feedback were compared with a multilevel linear regression model.

**Results:**

The mean mattress compression depth varied from 24.6 to 47.7 mm, with percentage of depletion from 31.2 to 47.5%. Both use of memory foam mattress (mean difference, MD 11.7 mm, 95%CI 4.8–18.5 mm) and use of backboard (MD 11.6 mm, 95% CI 9.0–14.3 mm) significantly minimized the mattress compressibility. Use of internal light sensor as source of feedback improved effective CC depth by 7–14 mm, compared with external accelerometer sensor.

**Conclusion:**

Use of a memory foam mattress and CPR backboard minimizes mattress compressibility, but depletion of compression depth is still substantial. A feedback device measuring sternum-to-spine displacement can significantly improve effective compression depth on a mattress.

**Trial registration:**

Not applicable. This is a mannequin-based simulation research.

## Background

Each year, approximately 209,000 people are treated for in-hospital cardiac arrest (IHCA) in the USA, with a survival rate of 22.7% in adults and 36.8% in children in 2014 [1]. Cardiopulmonary resuscitation (CPR) is a critically important treatment for cardiac arrest. High-quality CPR saves lives and directly influences patient outcomes [2–5]. Chest compression (CC) depth is highly associated with patient survival and good neurological outcomes [4, 6, 7]. However, the quality of CPR provided in both simulated [8] and real [9, 10] cardiac arrest events is often inadequate.

Providing CPR on a mattress further compromises CC depth compliance during IHCA. Previous studies investigating mattress deflection on intensive care unit (ICU) mattresses in both mannequin-based [11, 12] and real patient [13] studies show that mattress compression occurs during CPR and can reduce the proportion of CCs given with adequate depth. Once mattress compression is adjusted for, the proportion of CCs with appropriate depth decreases drastically [13, 14]. Furthermore, the total vertical hand movement is significantly larger than sternum-to-spine compression depth when CPR is performed on a mattress [12]. The additional motion and increased workload causes provider fatigue, which potentially impacts quality of CPR during the management of IHCA.

The use of a backboard underneath the patient during resuscitation has been shown to partly attenuate the compression effect of the mattress. Backboard use reduces the additional vertical hand movement of CPR providers and mattress compression depth [12], resulting in improved CC depth and percentage of CC with adequate depth [12, 15–19]. Other techniques that increase the rigidity of the mattress, such as the use of a mattress compression cover and a vacuum pump, have also shown to improve the effective compression depth [20]. Mattress compressibility has primarily been discussed as it relates to development of pressure sores in critically ill patients [21]. For this reason, it has been felt that compressible mattresses were potentially preferable for critically ill patients. It is vital, therefore, that we understand the relative risks of a very compressible mattress on CPR quality so the relative risks and benefits with respect to pressure sores and CPR quality can be determined. Some new ICU mattresses have memory foam to reduce overall compressibility. The effect of memory foam on CC depth has not been described.

Although the use of real-time feedback has been shown to improve quality of CPR in both training and clinical environments [8, 22], the use of single force and deflection sensor fails to adjust mattress compressibility and may overestimate compression depth when CPR is performed on a mattress. This may compromise effective compression depth [11, 13]. No prior studies have examined the impact of feedback source on CC depth when provided in different mattress contexts.

In this study, we aim to (i) quantify the amount of depletion when CPR is performed on two different ICU mattresses, with or without use of backboard; (ii) explore factors (i.e., mattress type, use of backboard) associated with mattress compression depth; and (iii) explore the effect of feedback sources on effective compression depth by pediatric intensive care unit (PICU) healthcare providers.

## Methods

We conducted a cross-sectional simulation-based study in the KidSIM Simulation Center at Alberta Children’s Hospital, an academic tertiary care healthcare facility in Calgary, Canada. Institutional review board approval was secured from the University of Calgary Conjoint Health Research Ethics Board and informed consent was obtained from all participants. We report our study in accordance with reporting guidelines for simulation-based research [23].

### Participants

Healthcare professionals from Alberta Children’s Hospital PICU were recruited to participate in the study. Inclusion criteria included (a) PICU healthcare providers (nurse, nurse practitioner, attending physician, respiratory therapist, resident, and fellow) and (b) receipt of Basic Life Support (BLS), Pediatric Advanced Life Support (PALS), and/or Advanced Cardiac Life Support (ACLS) certification within the past 2 years. Participants were excluded if they were unable to perform CC due to physical and/or medical reasons.

### Outcome measures

**Fig. 1:**
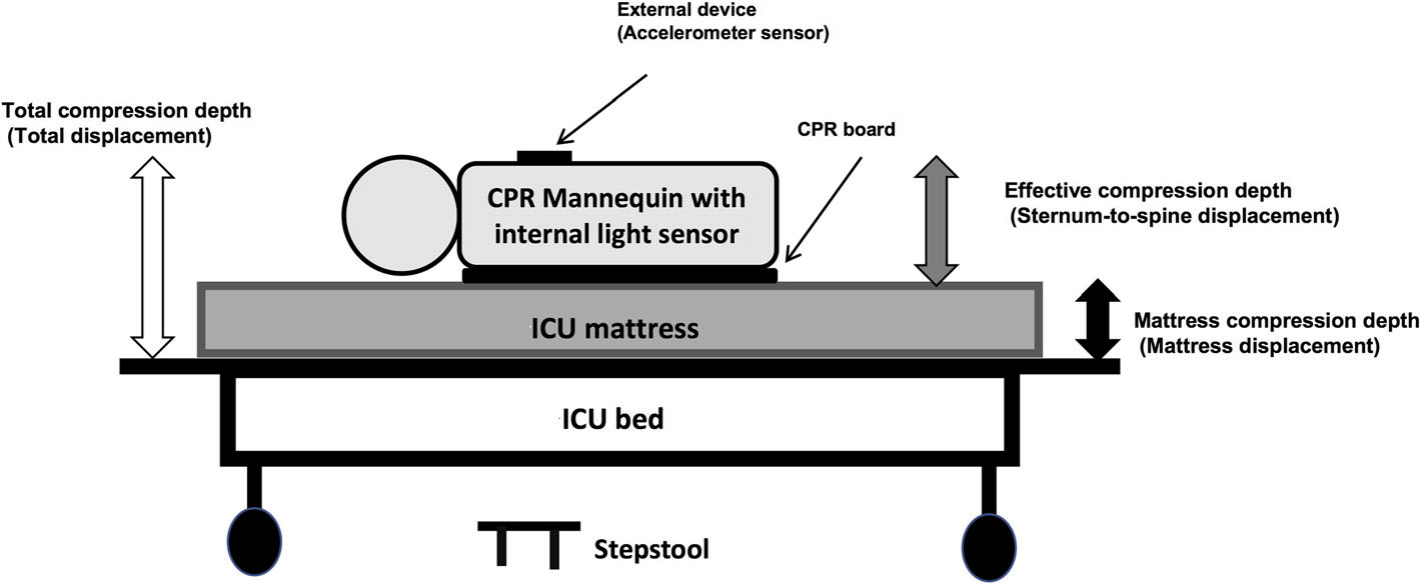
Description of study setting. Legend external device—measuring total displacement (effective compression depth+mattress compression depth); internal light sensor—measuring sternum-to-spine displacement of the mannequin (effective compression depth); mattress compression depth = total compression depth—effective compression depth

We used Laerdal Resusci Anne QCPR™ mannequin and the Laerdal CPR Meter™ to concurrently collect data for CC depth. The Laerdal Resusci Anne QCPR™ mannequin (internal device) measures the absolute sternum-to-spine displacement of the mannequin with a light sensor located inside, representing actual CC depth provided to the mannequin (i.e., *effective compression depth*). The Laerdal CPR Meter™ (external device) is an accelerometer sensor-based device placed on the chest of the mannequin during CC, which measures the total vertical hand movement delivered by the healthcare providers during CPR. The total vertical movement is the sum of effective compression depth and the amount of mattress compressibility (i.e., *total compression depth*; see Fig. 1). The primary outcome measure is the amount of mattress compression that occurred during CPR (i.e., *mattress compression depth*), calculated by the difference in measured CC depth between the mannequin’s internal device and the external device. The secondary outcome is the *effective compression depth* measured by the internal device.

### Study procedures

All participants completed a demographic survey upon recruitment into the study. Participants performed CC on a Laerdal Resusci Anne QCPR™ mannequin (weight = 8.5 kg) placed on an ICU bed (Hill-Rom 1000™ medical surgical bed) in each of the following four conditions: (i) on a typical ICU mattress (Advance 1000™ foam) only, (ii) on a typical ICU mattress with a CPR backboard, (iii) on a memory foam ICU mattress (Hill-Rom Accumax Quantum™ VPC Mattress) only, and (iv) on a memory foam ICU mattress with a CPR backboard. Participants were asked to perform high-quality CPR with a compression depth of 5–6 cm, at a rate of 100–120 per minute, with full chest recoil between compressions, in compliance with 2015 CPR guidelines [24–26]. Participants repeated each scenario twice and received real-time visual feedback for CPR quality provided by one of two different sources: (a) Laerdal CPR Meter™ (external device: accelerometer sensor) and (b) Laerdal Skillreporter™ (internal device: light sensor), in a random order generated by an online random number generator (8 min of chest compression in total). Between two 1-min chest compression sessions, each participant had opportunity to rest and rehydrate for 3–5 min to avoid fatigue.

We standardized the simulation-based research environment to reduce risk of bias [27]. We utilized the ICU mattresses with same size (213.4 cm × 90.2 cm × 15.2 cm) for each participant. The CPR backboard (hard plastic; 53.5 cm × 38 cm × 1 cm) used in conditions (ii) and (iv) was placed in a longitudinal orientation with the long axis of the backboard stretching down the length of the mattress. In all four conditions, the ICU mattress was placed on a bed at a fixed height of 75 cm. Participants provided compressions on a 23-cm high stepstool to optimize CC depth [28]. The study was conducted in a quiet room with no external stimuli. There were no observers in the room other than one or two research assistants. Participants were allowed to see the summary of their CPR performance at the end of each 2-min cycle.

### Sample size

Sample size estimation assumed that the main effects of memory foam mattress and backboard on mattress compression depth would comprise two comparisons. Allowing for Bonferroni adjustment, a significance level of 0.025 and power of 0.8 were used. We used individual chest compression as the unit of measure with each participant providing at least 100 compressions in each session. Assuming a standard deviation of 10 mm in compression depth, a total of 16 participants would detect a difference of 10 mm in mattress compression depth, even with a high correlation (*ρ* = 0.8) between repeated measures. The sample size allows us to detect a difference of 7 mm in effective compression depth with a significance level of 0.05 and power of 0.8.

### Statistical analysis

All analyses were conducted with R software (version 3.3.2, available at www.r-project.org) with “lme4” package [29]. To quantify the amount of mattress compressibility, we used data when internal device was used as a source of feedback (measuring effective CC depth). We summarized compression depth collected by an external device and an internal device as well as their differences with descriptive statistics (mean and standard deviation). Percentage of depletion was calculated (difference divided by total compression depth) in all four scenarios. To evaluate the effect of memory foam mattress use and backboard use on mattress compression depth, we use the same data to conduct mixed effect linear regression analyses. To evaluate the effect of feedback source on effective compression depth, we conducted a mixed effect linear regression analysis with random intercept and slopes, adjusting for use of backboard and types of mattress, with all data collected.

## Results

### Study population

Data from total of 12,101 individual compressions were analyzed. Compressions provided by 16 participants were included in the analysis, each of whom performed CPR for all four study conditions with two different sources of feedback. Three males (19%) and 13 females (81%) participated, including 3 physicians (19%), 10 nurses (62%), and 3 respiratory therapists (19%). All participants had Basic Life Support (BLS) and Pediatric Advanced Life Support (PALS) certification within the past 2 years and were actively involved in simulation-based training and/or research.

### Amount of chest compression depletion

**Fig. 2:**
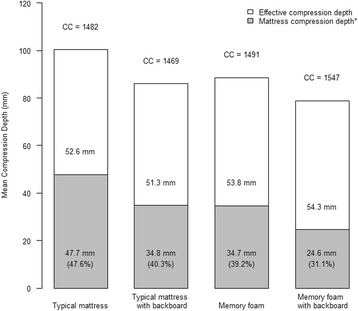
Mean chest compression depth in four study conditions. Legend: *percentage represents proportion of mattress compression depth over total compression depth. CC number of compression

When spine-to-sternal displacement was used a source of feedback, all healthcare providers performed guideline compliant chest compression (mean effective compression depth 51–54 mm). The mattress compression depth was 47.7 ± 18.7 mm (47.7%) [mean ± SD (percentage of depletion)] with typical mattress only, 34.8 ± 11.7 mm (40.3%) when the typical mattress was used with a CPR backboard, 34.7 ± 5.4 mm (39.2%) when a memory foam mattress was used, and 24.6 ± 5.5 mm (31.1%) when a memory foam mattress was used with a CPR backboard (see Fig. 2).

### Effect of foam mattress and use of backboard on depleted depth

Using a memory foam mattress decreased the mattress compression depth by 11.7 (95% CI 4.8–18.5) mm. The effect of a memory foam mattress was more pronounced without use of CPR backboard (13.3, 95% CI 6.4–20.2 mm) compared to with CPR backboard use (10.0, 95% CI 3.2–16.9 mm).

Use of a CPR backboard decreased the depleted compression depth by 11.6 (95% CI 9.0–14.3) mm. The effect of backboard use was greater with a typical mattress (13.3, 95% CI 10.6–15.9 mm) compared to memory foam mattress (10.0, 95% CI 7.4–12.7 mm) (see Table 1).

**Table 1:** Effect of backboard use and memory foam on mattress compression depth

Mattress compression depth (mm)	Mean (95% confidence interval)			
	No backboard	Backboard	Backboard benefit	Backboard mean benefit main effect
Typical mattress	47.9 (40.5, 55.3)	34.6 (28.6, 40.7)	13.3 (10.6, 15.9)	11.6 (9.0, 14.3)
Memory foam	34.6 (28.6, 40.7)	24.6 (22.5, 26.7)	10.0 (7.4, 12.7)	
Memory foam benefit	13.3 (6.4, 20.2)	10.0 (3.2, 16.9)	NA	NA
Memory foam mean benefit main effect	11.7 (4.8, 18.5)		NA	NA

Presented are the mean adjusted values from a mixed effects linear model. Fixed effect: mattress type, use of CPR backboard and interaction between mattress type and use of CPR backboard. Random effect: intercept, mattress type, and use of CPR backboard. Goodness of fit: marginal *R* ^2^ = 0.33, conventional *R* ^2^ = 0.82

*NA* not applicable

### Effect of different sources of feedback on effective compression depth

When an anterior device was used as the source of feedback, participants failed to conduct guideline compliant CPR in all four scenarios (mean CC depth 37.9–46.2 mm). When sternal-to-spine displacement was used to guide CPR quality, participants conducted guideline-compliant effective CPR (mean CC depth 51.3–54.3 mm) in all four scenarios. The estimated effect of using an internal device on effective compression depth was 14.3 (95% CI 12.0–16.5) mm for a typical PICU mattress only, 8.7 (95% CI 6.5–11.0) mm on a typical PICU mattress with a CPR backboard, 13.0 (95% CI 10.7–15.3) mm on a memory foam mattress only, and 7.5 (95% CI 5.2–9.8) mm on a memory foam mattress with a CPR backboard (see Table 2).

**Table 2:** Effect of source of feedback on effective compression depth

Effective compression depth (mm)	Mean (95% confidence interval)		
	Source of feedback		
	Anterior sensor	Internal measure	Difference
Typical mattress only	37.8 (34.1, 41.5)	52.1 (50.1, 54.1)	14.3 (12.0, 16.5)
Typical mattress + backboard	42.9 (39.5, 46.3)	51.7 (49.8, 53.5)	8.7 (6.5, 11.0)
Memory foam mattress only	41.2 (37.9, 44.4)	54.2 (52.3, 56.1)	13.0 (10.7, 15.3)
Memory foam mattress + backboard	46.3 (43.2, 49.3)	53.7 (52.0, 55.5)	7.5 (5.2, 9.8)

Presented are the mean adjusted values from a mixed effects linear model. Fixed effect: mattress type, CPR backboard use, feedback source, interaction between feedback source and mattress type, and interaction between feedback source and CPR backboard use. Random effect: intercept, mattress type, CPR backboard use, and feedback source. Goodness of fit: marginal *R* ^2^ = 0.43, conventional *R* ^2^ = 0.80

## Discussion

This is the first study to demonstrate the effect of memory foam mattress on mattress compressibility during CPR and reinforce the limitation of single accelerometer sensor for feedback on chest compression depth, to our knowledge. We demonstrated that providing CC on an ICU mattress leads to a significant amount of mattress compressibility. The combined use of memory foam mattress and a CPR backboard resulted in the least amount of mattress compression depth. Using devices to measure sternum-to-spine displacement as a source for real-time feedback improved effective compression depth, compared with single anterior accelerometer sensor.

Previous studies have demonstrated the importance of high-quality CPR on survival and neurological outcome of patients with cardiac arrest. In adult out-of-hospital cardiac arrest victims, even a slight increase in CC depth (5 mm) was associated with increased survival to hospital discharge (adjusted odds ratio, OR 1.29) and favorable neurologic outcomes (adjusted OR 1.30) [4]. In pediatric cardiac arrest, guideline-compliant compression depth (CC depth > 51 mm) was associated with improved 24-h survival [7]. However, most healthcare providers fail to provide guideline-compliant CPR during real [3, 4, 7, 9, 10] or simulated [8, 30] resuscitation events.

Mattress compressibility further compromises the CC depth of healthcare providers managing cardiac arrest. Noordergraaf et al. reported that the total vertical hand movement is larger when CPR was conducted on mattress [12], which further increased workload and fatigues of compression providers. Mattress compressibility has been shown to decrease the effective CC depth and percentage of compression meeting guideline in CPR-certified healthcare providers [31]. Previous mannequin-based studies have demonstrated the mattress compressibility ranged from 4 mm (ED stretcher mattress) to 13 mm (ICU mattress) depending on mattress types [13]. In our study, we found the mattress compression depth was as high as 24.6 mm, with 31.1% of CC depth depleted by mattress compressibility, even in the best-case scenario. This means providers need to push approximately one-third deeper than the guideline-recommended depth, to ensure CC provided is guideline-compliant. CPR providers should be aware that providing chest compressions on mattresses feels different from those taught during a conventional BLS course, where compressions are typically done on the floor or hard surfaces. CPR training should allow healthcare providers to practice chest compressions with proper real-time feedback on the mattress that patients are typically placed on in their relevant clinical unit.

Given the importance of high-quality CPR, it is critical to identify strategies to reduce mattress compressibility during resuscitation events. In our study, we found the use of a memory foam mattress significantly improves mattress compressibility by 11.7 mm, which could potentially result in a significant difference in patient outcomes. Previous research on techniques that increase the rigidity of the mattress have shown similar outcomes. Oh et al. reported an innovative mattress with several tubes inserted for deflation improved CC efficiency (proportion of effective depth over total depth) from 42 to 81% [32]. Other methods like mattress compression covers and vacuum pumps also significantly decreased mattress compression depth and increased the efficiency of chest compression [20]. We also found that the application of a CPR backboard reduces mattress compressibility by a significant depth (11.6 mm) in our study. Our results are consistent with previous studies that demonstrate reduction in vertical hand movement of CPR providers and mattress compressibility [12] with use of a CPR backboard. CPR backboard use does not necessarily guarantee effective compression depth. Some previous studies showed that the utilization of a CPR board increased CC depth and resulted in guideline-compliant depth [16, 18], while other studies reported CC depth well below guideline [14, 33], since the effective compression depth was influenced by numerous other factors, such as timing and frequency of training [34–36] and the use of feedback [8, 22, 37, 38].

The use of real-time feedback devices has played an important role in improving CPR quality [8, 22, 37, 38]. Our study indicates that the source of feedback may influence the quality of CPR delivered. The use of single anterior force sensor results in misinterpretation of CC depth when CPR is performed on the mattress. Nishisaki et al. reported that proportion of CC meeting CPR guidelines was only 31.1% with overestimation of 13 mm in CC depth when adjusting for mattress compressibility [13]. Similarly, Hellevuo et al. demonstrated that real-time feedback from single sensor overestimated the effective CC depth by 10 mm when CPR was performed by practicing paramedics [31]. In our study, we found healthcare providers failed to achieve guideline-compliant effective compression depth in all scenarios when an anterior sensor was used as a source of feedback. When using internal device, which measures sternum-to-spine displacement, the effective compression depth was improved for 7–14 mm. Although the sample size estimation is primarily based on mattress compression depth, even in the case where there was the smallest effect demonstrated (i.e., memory foam mattress + backboard), the lower limit of the 95% confidence interval (5.2 mm) remained clinically significant (greater than 5 mm [4]). Since our patients will not have an internal device built inside, accelerometer is still the main technology for CPR feedback used in real patient resuscitation. However, healthcare providers should preferentially utilize dual sensor devices, where an additional accelerometer sensor put on the back of the patient adjusts for mattress compressibility. If a single anterior sensor device is used, healthcare providers should not purely rely on the feedback information entirely, because the feedback provided is an overestimation of effective compression depth. Critically ill patients are at risk of pressure sores, and the type of mattress that they lie on can increase that risk [21]. Therefore, one must also consider both CPR quality and pressure sore risk when selecting mattresses in an ICU setting.

### Limitations

Our study has several limitations. First, we focused on the mattress compressibility in a simulated environment with only one type of mannequin. In the real clinical world, the factors associated with mattress compressibility might be more complicated, such as patient size/weight, size, the orientation of the backboard [19], and chest compliance. The weight of the mannequin torso used in the study was much lower than the real patients, which could lead to potential bias in estimating mattress compressibility. Second, we did not directly link the mattress compression depth with effective compression depth in our study. However, we believe reducing mattress compressibility will result in decreased fatigue level of CPR providers, and thus leading to improved effective compression depth. Third, we chose only two different types of mattress used in the ICU. This limits generalizability of the study. Fourth, unlike previous studies using mechanical devices, we recruited CPR-certified healthcare providers to provide CC. This could introduce some variability in quality of compressions. However, we provided real-time feedback to participants to minimize the variability of compression quality. In addition, this better represents the variance encountered in real clinical encounters and thus improves the generalizability. Fifth, the majority of participants were female in this study, thus possibly influencing the generalizability of the conclusion. Last, but not the least, we were not able to examine chest recoil due to technical limitations.

## Conclusion

Chest compression depth is significantly depleted when CPR is performed on an ICU mattress. Mattress firming technology should be considered for patients at high risk for cardiac arrest. A CPR backboard should always be used when managing cardiac arrest. When real-time feedback is used, healthcare providers should consider devices that measures sternum-to-spine displacement (i.e., dual accelerometer sensor) to improve effective compression depth.

## Abbreviations

ACLS: Advanced Cardiovascular Life Support
BLS: Basic Life Support
CC: Chest compression
CPR: Cardiopulmonary resuscitation
ICU: Intensive care unit
IHCA: In-hospital cardiac arrest
MD: Mean difference
OR: Odds ratio
PALS: Pediatric Advanced Life Support
PICU: Pediatric intensive care unit
